# Deoxynivalenol and T-2 Toxin as Major Concerns in Durum Wheat from Italy

**DOI:** 10.3390/toxins14090627

**Published:** 2022-09-08

**Authors:** Miriam Haidukowski, Stefania Somma, Veronica Ghionna, Maria Teresa Cimmarusti, Mario Masiello, Antonio Francesco Logrieco, Antonio Moretti

**Affiliations:** Institute of Sciences of Food Production, National Research Council (ISPA-CNR), via Amendola 122/O, 70126 Bari, Italy

**Keywords:** *Fusarium*, trichothecenes, mycotoxin occurrence, species distribution, geographical areas

## Abstract

Fusarium Head Blight is a devastating disease of wheat caused by a complex of *Fusarium* species producing a wide range of mycotoxins. *Fusarium* species occurrence is variable in different geographical areas and subjected to a continuous evolution in their distribution. A total of 141 durum wheat field samples were collected in different regions of Italy in three years, and analyzed for *Fusarium* species and related mycotoxin occurrence. Mycotoxin contamination varied according to year and geographical origin. The highest mycotoxin contamination was detected in 2014. Deoxynivalenol was detected with an average of 240 µg/kg only in Central and Northern Italy; and T-2 and HT-2 toxins with an average of 150 µg/kg in Southern Italy. Approximately 80% of samples from Southern Italy in 2013/2014 showed T-2 and HT-2 levels over the EU recommended limits. *Fusarium graminearum* occurred mostly in Northern Italy, while *F. langsethiae* occurred in Southern Italy. These data showed that a real mycotoxin risk related to *Fusarium* exists on the whole in Italy, but varies according with geographical areas and environmental conditions. Consistent monitoring of *Fusarium* species and related mycotoxin distribution on a long period is worthwhile to generate more accurate knowledge on *Fusarium* species profile and mycotoxins associated and better establish the climatic change impact on wheat *Fusarium* epidemiology.

## 1. Introduction

Fusarium Head Blight (FHB) of wheat is an economically devastating disease worldwide that can cause multibillion-dollar losses to world agriculture [1]. The disease is caused mainly by a complex of *Fusarium* species, most of which are able to produce a wide range of mycotoxins, trichothecenes above all, that can be accumulated in wheat kernels at maturity and may be a serious concern for both human and animal health, since they interfere with protein synthesis and cause a wide range of toxic effects, including vomiting, intestinal inflammation and gastrointestinal hemorrhage [2].

Among *Fusarium* species, the main etiological agent of FHB worldwide is *Fusarium graminearum sensu stricto* [3], a globally widespread species that produces mainly deoxynivalenol (DON), nivalenol (NIV) and zearalenone (ZEA) [2]. Together with *F. graminearum s.s.*, *F. culmorum* has also been frequently reported as a main pathogen of wheat worldwide [4], being able to produce the same mycotoxins as *F. graminearum* [2]. Yield and quality losses are particularly important when both species induce FHB, which develops from infection at anthesis and spreads until grain harvest, causing grain contamination with mycotoxins, such as type B trichothecenes, ZEA and fusarins [5]. *Fusarium poae*, *F. langsethiae*, *F.sporotrichioides*, *F. avenaceum*, *F. tricinctum* and *F. acuminatum* are reported frequently among the other detected species in wheat kernels [6,7]. *Fusarium poae* is a species of increasing importance as it is involved in FHB in many countries such as Argentina, Canada, Finland, Belgium, Germany, Switzerland, Hungary, Slovakia, Italy, Ireland and the United Kingdom [8]. The prevalence of *F. poae* in wheat reported in recent years is surprising because the species was believed to be less aggressive than other FHB pathogens [6,8]. On the other hand, *F. avenaceum* is dominant throughout regions with a climate that is more cool and wet, such as Northern, Central Europe and Canada [9]. However, this species often occurs on cereals isolated in warm areas, as reported by [10]. The two closest phylogenetically related species to *F. avenaceum*, *F. tricinctum* and *F. acuminatum*, are usually less widespread and relatively weak pathogens than *F. avenaceum*, and they occur more frequently in regions with a cool or temperate climate [11].

Non-ambiguous taxonomic identification of *Fusarium* species using a morphological approach is often very difficult because of their high similarity. For instance, *F. avenaceum* is often confused with *F. acuminatum* [12,13]; *F. tricinctum* can be misidentified as *F. sporotrichioides*; *F. poae* with *F. langsethiae* [13,14]; and *F. culmorum* is similar to *F. sambucinum* [15].

The distribution and predominance of the different *Fusarium* species involved in FHB development are mostly related to agronomic practices, such as cropping sequence, soil tillage, sowing on untilled soil, use of nitrogen fertilizers and host genotype, as well as to competition among species [16]. Moreover, the species profile is subjected to a continuous evolution in distribution, due to climate change [17], and needs to be constantly monitored. The combinations of the different species occurring on wheat heads and the impact of the environment on such pathogen complexes are not well understood. Therefore, it is of paramount importance to know the exact FHB pathogens at a particular site, since the effects of environmental conditions on FHB development may differ considerably between FHB pathogens [18], also in response to different fungicides used to control FHB [19], and FHB pathogens differ in the ability to produce mycotoxins.

The most important mycotoxins associated with durum wheat are trichothecenes and zearalenone. However, other emerging mycotoxins such as beauvericin, enniatins, fusaproliferin, and moniliformin have also been detected in wheat kernels [20].

Trichothecenes are classified into two main groups: type-A, including HT-2 toxin (HT-2) and T-2 toxin (T-2), and type-B, including nivalenol (NIV), 4-acetyl-nivalenol, deoxynivalenol (DON), 3- and 15-acetyl-deoxynivalenol (3-AcDON and 15-AcDON). Type A trichothecenes are produced by several *Fusarium* species involved in FHB such as *F. langsethiae*, *F. poae*, and *F. sporotrichiodes* [21]. *Fusarium langsethiae* and *F. sporotrichioides* are the main T-2- and HT-2-producing species. Deoxynivalenol is the most important among the type B trichothecenes, due to its natural and widespread occurrence in the crops at high levels. The main fungal species responsible for DON contamination are *F. graminearum*, *F. culmorum* and *F. croockwellense* [2]. Deoxynivalenol is frequently found in association with zearalenone (ZEA) [22], an estrogenic resorcylic acid lactone, mostly produced by *F. graminearum*, *F. culmorum* and *F. crookwellense* [2]. Contamination is prevalent in temperate climates when relatively cool temperatures and high humidity coincide with the flowering and early kernel filling stages of the grain [23]. Since it is not completely possible to prevent the synthesis of mycotoxins, national and international authorities have adopted regulatory limits and guidelines to monitor mycotoxin levels in various food and feed products [24,25,26].

In particular, the limit of 1750 μg/kg has been fixed for DON in unprocessed wheat, oats and maize cereals [24], whereas the limit of 100 μg/kg has been fixed for zearalenone in unprocessed durum wheat. Regulatory limits have been discussed by the European Commission, considering the sum of T-2 and HT-2 in cereals and cereal products. The latest proposal in this regard is 100 μg/kg for unprocessed cereal [26].

In recent years, worldwide climate changes are leading to an ever-changing profile of the typical dominant *Fusarium* species on cereals, which can also result in dramatic variability in associated mycotoxins. Therefore, nowadays, it is very important to monitor and to identify *Fusarium* species and their mycotoxins on cereal crops, in order to promptly assess the mycotoxicological risk and develop control strategies to minimize the risk.

The aim of this work was to monitor the occurrence of *Fusarium* species and associated mycotoxins on durum wheat collected in three different geographical macro-areas of Italy: Northern, Central, and Southern. Sampling was carried out during three different years (2013, 2014 and 2015) with the aim of evaluating the effects of possible changes in environmental conditions in different seasons. An understanding of the species and mycotoxin profile of FHB pathogens is essential for reliable disease predictions and management, and for reducing the food risks associated with mycotoxins.

## 2. Results

### 2.1. Mycotoxin Contamination of Wheat Samples from Different Geographical Italian Areas

**Figure 1:**
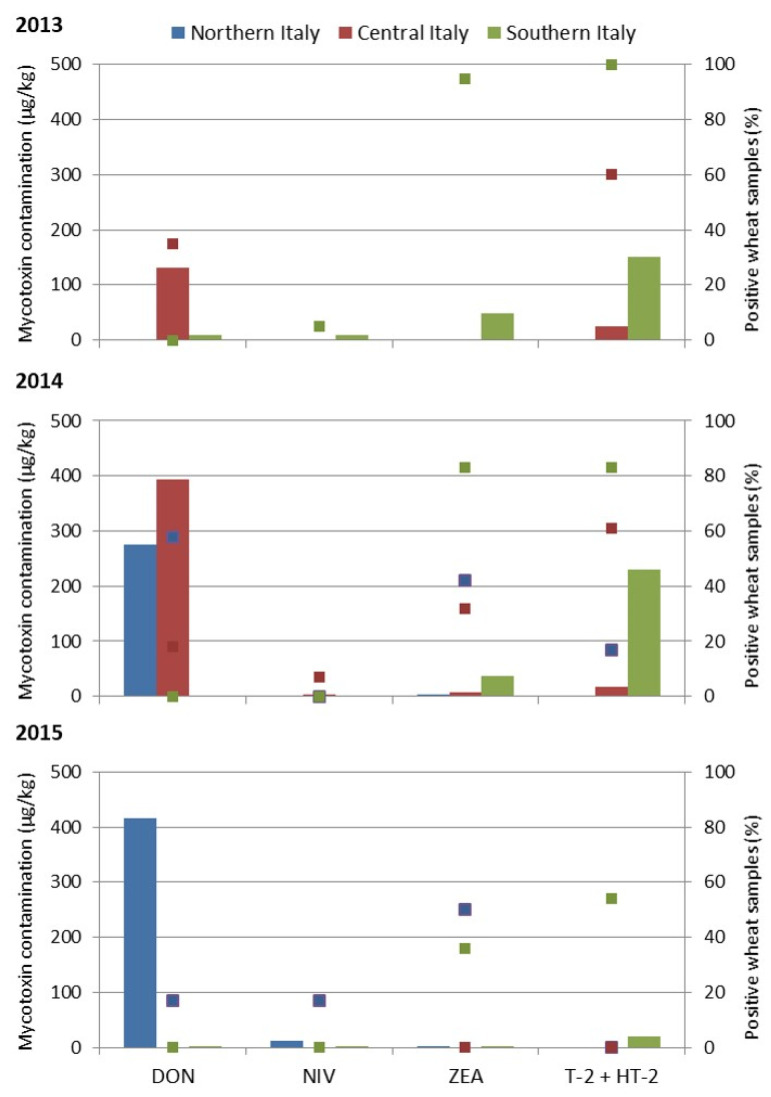
Mycotoxin contamination of wheat samples collected in Italian geographical areas. The amount of deoxynivalenol (DON), nivalenol (NIV), and zearalenone (ZEA) and the sum of T-2 and HT-2 toxins, expressed in µg/kg as bars, and the percentage of positive wheat samples (points) are shown for each geographical area in three years. In 2013, no samples from Northern Italy were collected and only DON and T-2 + HT-2 were analyzed in Central Italy.

The mycotoxin mean values calculated on total analyzed wheat samples were graphically reported in Figure 1, with the percentage of the positive contaminated samples.

The average of contamination by DON, NIV, ZEA and T-2 + HT-2 mycotoxins on the total samples was reported separately for Northern (NI), Central (CI) and Southern Italy.

In 2013, DON was present mostly in Central Italy, with an average value of 132 µg/kg; NIV was rare; ZEA was detected in 95% of wheat samples from Southern Italy, but with a mean value of 49 µg/kg; T-2 + HT-2 toxins were present in Central Italy and Southern Italy (60 and 100% of contaminated samples) with values of 24 and 151 µg/kg, respectively.

In 2014, wheat from Northern and Central Italy was mostly contaminated by DON, at average values of 275 and 393 µg/kg, respectively. Additionally, in this year, NIV was rare, while all Italian wheat samples were contaminated by ZEA, although with low values of 4, 8 and 37 µg/kg in Northern, Central and Southern Italy, respectively. Similarly, T-2 and HT-2 were detected in all Italian samples, with a high mean value in Southern Italy, 231 µg/kg.

In 2015, high contamination by DON was detected in samples from Northern Italy, with 417 µg/kg as the average value. The other mycotoxins were detected at negligible values in all Italian regions.

The rate of wheat samples contaminated by mycotoxins and the statistical data of the detected mycotoxins evaluated on positive samples are shown in Table 1. In this way, we can better appreciate the risk derived from contaminated wheat sample consumption.

In 2013, wheat samples from Central Italy were contaminated by DON (35%), up to 2230 µg/kg, with a mean of 376 µg/kg (one sample exceeded EU legal limit); and by T-2 and HT-2 toxins, up to 123 µg/kg, with two samples over the EU limits. Nivalenol and ZEA were not analyzed. In the same year, in the samples from Southern Italy, DON was not detected, NIV was detected only in one sample, while 95% of the samples were contaminated by ZEA, up to 297 µg/kg, with three of them exceeding the EU legal limits. T-2 and HT-2 toxins, up to 335 µg/kg, with a mean value of 154 µg/kg (Table 1) were detected in all the wheat samples; the values exceeded the EU limits in 17 out of 20 samples.

In 2014, DON was detected in Northern Italy (58% of samples with a mean value of 462 µg/kg), and in Central Italy (18% of samples with a mean value of 2227 µg/kg, and one value over the EU limits); NIV was detected only in two samples from Central Italy (with a mean value of 56 µg/kg); ZEA contamination was detected in 42, 32 and 83% of wheat samples, with mean values of 9, 24 and 49 µg/kg, in Northern, Central and Southern Italy, respectively; three samples in Southern Italy exceeded the EU limits. The samples contaminated by T-2 and HT-2 toxins were 17, 61 and 83%, with mean values of 11, 28 and 310 µg/kg in Northern, Central and Southern Italy, respectively; in Southern Italy, the values of 18 samples out of 24 were over the EU limits (Table 1).

In 2015, almost all the wheat samples were free from mycotoxins. In Northern Italy, one sample was contaminated by DON, at a very high level (2500 µg/kg), one by NIV (76 µg/kg), and three by ZEA (mean value of 6 µg/kg). In Southern Italy, 36% of samples were contaminated by ZEA with values of 5 µg/kg; and T-2 and HT-2 toxins were detected in 54% of samples with a mean value of 28 µg/kg (Table 1).

### 2.2. Fungal Contamination of Italian Wheat Samples

Mycological analyses of wheat kernels collected in Italy during three years (2013, 2014 and 2015) were carried out to evaluate the overall fungal contamination and, in particular, the contamination by toxigenic fungi.

Total fungal contamination detected on all wheat samples was very high, with mean values of 94, 93 and 88% in 2013, 2014 and 2015, respectively. Any wheat sample was contaminated by *Aspergillus* or *Penicillium* species. *Alternaria* species were always present in wheat samples, with variable values from 14 to 86%.

The percentage of kernel contaminated by *Fusarium* species ranged between 0 and 8% in Southern Italy in all three years and in Central Italy in 2015; between 0 and 38% in Central Italy in 2014; between 0 and 12% in Northern Italy in 2014. Very high *Fusarium* contamination was detected in Northern Italy in 2015, ranging between 25 and 76%, with a mean value of 51%.

### 2.3. Fusarium Species Distribution in Italian Wheat Samples

**Figure 2:**
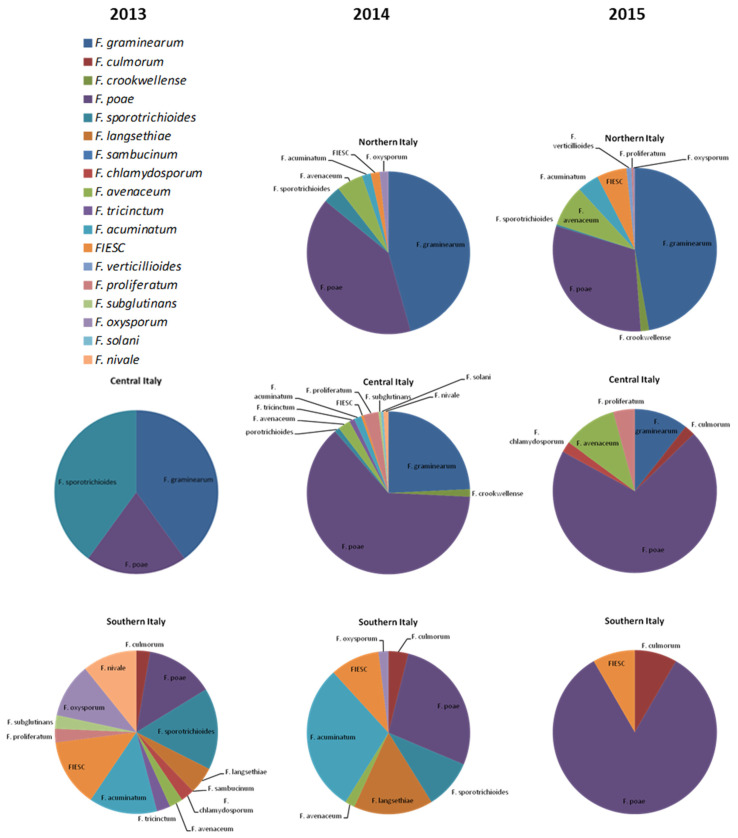
Distribution of *Fusarium* species isolated from wheat samples collected from different Italian geographical areas during the crop seasons 2013, 2014 and 2015.

Several *Fusarium* species were identified from wheat kernels, most of them able to produce mycotoxins. In Figure 2, the distribution of *Fusarium* species for Northern, Central and Southern Italy was reported for each year.

In Northern Italy, the predominant species were *F. graminearum* (46 and 47% in 2014 and 2015, respectively) and *F. poae* (47 and 31% in 2014 and 2015, respectively). The remaining rate was constituted by *F. sporotrichioides*, *F. avenaceum*, *F. acuminatum* and strains belonging to *Fusarium Incarnatum-Equiseti* Species Complex (FIESC).

In Central Italy, *F. poae* was the most spread species, with values of 20, 63 and 70% in 2013, 2014 and 2015, respectively, followed by *F. graminearum*, detected as 40, 24 and 11%.

In Southern Italy, *F. graminearum* was totally absent in the three years, while *F. culmorum* and *F. langsethiae* strains, rare in the rest of Italy, were identified. *Fusarium poae*, *F. sporotrichioides*, *F. acuminatum* and FIESC were the most detected species, with comparable values. To a lesser extent, *F. avenaceum*, *F. oxysporum, F. chlamydosporum*, *F. proliferatum* were also detected.

### 2.4. Mycotoxin Risk Hypothesized in Wheat Samples from Different Geographical Italian Areas

**Figure 3:**
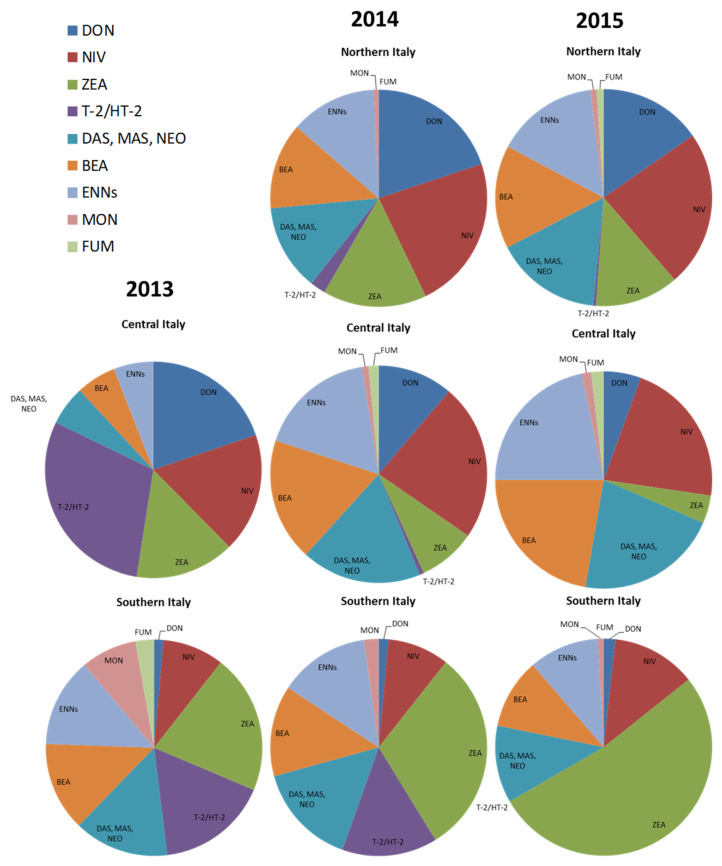
Supposed potential mycotoxin contamination risk in durum wheat, in different Italian geographical areas, based on *Fusarium* species detected in three years. The mycotoxins considered for the responsible *Fusarium* species were: deoxynivalenol (DON), nivalenol (NIV), zearalenone (ZEA), T-2 and HT-2 toxins (T-2/HT-2), diacetoxyscirpenol (DAS), monoacetoxyscirpenol (MAS), neosolaniol (NEO), beauvericin (BEA), enniatins (ENNs), moniliformin (MON), and fumonisins (FUM).

Based on the knowledge of the *Fusarium* producing species for each mycotoxin (reported in Table 2) and on the number of strains belonging to the different toxigenic *Fusarium* species detected in this study in Italian regions, we arranged the data of potential risk of mycotoxin contamination in Italian durum wheat (Figure 3).

The graphs show a wide risk of multi-mycotoxin contamination in Italian durum wheat. Many mycotoxins are always present in the three years and in the different geographical areas, except for Northern Italy in 2013, but it was possible to distinguish specific risk in Italy. In particular, DON risk is high in Northern and Central Italy, while it is negligible in Southern Italy. Nivalenol risk is always present, although more in Northern and Central Italy and in lower percentage in Southern Italy. On the contrary, ZEA, always present too, is more evident in Southern Italy. The risk of T-2 and HT-2 toxins is negligible in Northern Italy, high in Southern Italy, and variable in Central Italy, depending on the years. The risk linked to the other minor mycotoxins, such as ENNs, BEA, DAS, MAS, and NEO, is also present in all the geographical areas and in all years. Negligible risk is related to MON and FUM in Italian durum wheat.

## 3. Discussion

This work provides key information about the contamination of Italian durum wheat by *Fusarium* species responsible for FHB and associated mycotoxins in the period 2013–2015. The results of this monitoring are reported focusing on three major geographical Italian areas, Northern, Central and Southern Italy, in order to evaluate the variation of mycotoxin contamination and *Fusarium* toxigenic species associated with the different climatic and environmental conditions that characterize the above-mentioned areas.

From this study, high variability in mycotoxin contamination of Italian durum wheat emerges over the years, confirming the high, well-known influence of environmental conditions on the spread of infections by toxigenic fungi [17,27]. Both 2013 and 2014 had more serious mycotoxin contamination levels than 2015, a year characterized by abnormal hot weather, in which contamination was negligible for all mycotoxins in Italy. Indeed, climate indicators characterize 2015 as among the hottest years of the last half century, with an average annual temperature anomaly of +1.58 °C, compared to the period 1961–1990, and cumulative annual rainfall below the climatological average of approximately 13% in Italy [28].

However, specific mycotoxins are mainly present in the different geographical areas, as DON in Northern and Central Italy and T-2 and HT-2 toxins in Southern Italy. Mycotoxin contamination was also evaluated taking into account the legal limits established by the EU. Although this study is based on wheat samples collected in the period 2013–2015, these data are very important because they show the *Fusarium* contamination of Italian durum wheat in a period for which, as far as we are aware, no information is available in the literature. Since the importance of continuous monitoring of fungal and mycotoxin contamination has always been highlighted, the data shown in this study need to be compared to both previous [29,30,31] and subsequent years, in order to evaluate eventual differences or changes in FHB species composition and mycotoxins.

If the levels of contamination are considered as average values on the total analyzed samples (Figure 2), Italian durum wheat contamination is not very high. However, when the data are evaluated on the positive samples, contamination is more worrisome (Table 1). Deoxynivalenol was absent in Southern Italy, but reached 58% of contaminated samples with values up to 9129 µg/kg in Northern and Central Italy. The maximum values established in EU legal limits were exceeded in four samples. On the contrary, T-2 and HT-2 toxins predominate in Southern Italy, contaminating up to 100% of samples, among which 17 and 18 samples out of 20 in 2013 and 2014, respectively, exceeded the EU recommended level of 100 µg/kg. These data demonstrate a rather worrying threat for food safety in the wheat chain, mostly in Southern Italy. Similarly, ZEA was detected everywhere, with approximately 15% of samples exceeding the legal limits in Southern Italy. On the contrary, NIV was rare in Italy in all three years, showing that it is not a threat for Italian durum wheat. These data underline the importance of mycotoxin monitoring in restricted areas to better realize the specific hazard deriving from contaminated wheat consumption.

A further consideration arising from the present study is that many samples were simultaneously contaminated by more than one mycotoxin, in agreement with other studies [32,33]. In particular, the co-occurrence of trichothecenes and ZEA is commonly found in cereals [32]. The concern of multiple mycotoxin contamination poses a further risk to animal and human health, because of their possible additive, if not synergistic, effects [34,35]; thus, more studies on mycotoxin toxicological interactions are strongly requested and stressed.

The most frequent species isolated and identified in this work are *F. graminearum s.s.*, *F. poae*, *F. avenaceum*, and *F. sporotrichiodes*, species which are currently reported as involved in FHB worldwide. As reported for mycotoxin contamination, also among *Fusarium* species distribution in Italy, it was possible to distinguish different frequencies associated with the geographical areas (Figure 3).

Overall, *F. graminearum* s.s. is most prevalent in Northern Italy, generally present in Central Italy depending on the year, and rare, if not absent, in Southern Italy. This perfectly reflects previous mycotoxin contamination reports on DON and agrees with previous studies demonstrating that DON is the most widespread toxin, with the highest level of contamination in the Emilia Romagna region [29]. On the other hand, *F. sporotrichioides* and *F. langsethiae* were mostly detected in Southern Italy, when very high contamination rates and levels were registered, except in 2015, in which fungal and mycotoxin contamination was slight and occasional, perhaps due to climatic conditions. On the contrary, the above-mentioned species were rare in Northern Italy in 2014 and 2015 and *F. sporotrichioides* was only slightly detected in Central Italy, mostly in 2013, as for T-2 and HT-2 toxin contamination. These data agree with the report of Infantino et al. [36] that observed a higher incidence (84.0%) of durum wheat samples positive for *F. langsethiae* from central and southern Italian regions.

*F. graminearum* contamination notoriously causes more concern because DON is often associated with and is well known in Northern Italy [37]. On the other hand, it is necessary to underline the growing threat of the heavy presence of T-2 and HT-2 in Southern Italy. *Fusarium langsethiae* has an optimum growth temperature in the range of 20–30 °C [38]. The very high contamination rate, up to 100%, of durum wheat samples, associated with the high concentration values, above the recommended levels in most of the samples, indicate that also establishing legal limits for these mycotoxins should be evaluated carefully, although the Report of European Food Safety Authority (EFSA) on T-2 and HT-2 contamination levels in Europe still ensures that there is not such a need yet [39]. The importance of this issue is not only due to the acute and chronic toxicity of two of the most toxic trichothecenes, with very high immunosuppressive and hematotoxic effects [21], but also due to their capability to be metabolized in modified mycotoxins in planta, with the subsequent risk of achieving their highest bioavailability, once ingested through the wheat by-products [40]. Although oat grain is the crop most affected by the accumulation of HT-2 and T-2 compared to wheat and barley [41], *F. langsethiae* has been reported as an important contaminant of wheat in Italy as evidenced by the report of Infantino et al. [36].

In addition, until recently, due to the indistinguishable morphological traits between *F. langsethiae* and *F. poae*, the latter species has often been overestimated, and includes the contamination of *F. langsethiae*, which was described as *Fusarium* powdery *poae* [42].

Among *Fusarium* species, *F. poae* was the most common species detected in all the Italian wheat samples, despite a very low NIV contamination. This result is the opposite to previous studies in Italy [30], in which NIV was detected ubiquitously at appreciable concentrations. However, the reported high frequency of *F. poae* here, coupled with the reduced occurrence of *F. graminearum* over the years, was already stated by Xu and Nicholson [6] in Europe, probably due to changing environmental conditions.

Moreover, the frequency of *Fusarium* species isolated in Southern Italy is evenly distributed unlike in Northern Italy. This underlines the higher risk in Southern Italy of the co-occurrence of multiple pathogens within an individual host, causing multiple fungal infections and mycotoxin contamination, thus also requiring better monitoring of minor mycotoxins. To evaluate this risk, the potential mycotoxin contamination risk in durum wheat, in different Italian geographical areas, based only on *Fusarium* species detected in three years, was evaluated and graphically shown in Figure 3. As reported, the supposed mycotoxin risk matches the effective detected contamination of DON and NIV, more present in Northern areas, and of T-2 and HT-2 toxins, mostly in Southern areas, while ZEA is spread all over the country. The main difference is the evaluated risk of minor mycotoxins, since the risk linked to ENNs, BEA, DAS, MAS and NEO is high in all the geographical areas and in all years. Although negligible, FUM risk is also detected in this study, consistent with recent studies on an increasing presence of *F. proliferatum* in durum wheat [43,44,45]. Usually, the surveys on mycotoxin contamination in cereals are mostly targeted towards major mycotoxins such as DON, NIV, ZEA, T-2 and HT-2. However, the hypothesized risk poses the problem of co-occurrence of different mycotoxins in wheat samples. Thus, the contamination by minor mycotoxins as well as the toxicological interactions among mycotoxins should not be disregarded.

The presence of *F. langsethiae* and T-2 and HT-2 toxin contamination in symptomless kernels of wheat is important for food and feed safety, because the lack of visual symptoms does not mean that the grains are free from these mycotoxins. This absence of symptoms of disease can be considered an additional factor of risk because of unaware exposure to T-2 and HT-2, which, among trichothecenes, are considered the most acutely toxic members. Moreover, despite the low levels detected, FUM can also occur in wheat, and therefore should be included in monitoring programs. Finally, due to the high risk of mycotoxin contamination in durum wheat, reported in our study, confirmed by the effective detection of T-2 and HT-2 toxins, more extended and consistent studies on *F. sporotrichioides* and *F. langsethiae* occurrence in Italian durum wheat are strongly recommended for a more accurate risk assessment related to these harmful mycotoxins and to provide more tools for management strategies aimed to mitigate *Fusarium* mycotoxins on durum wheat in Italy.

## 4. Materials and Methods

### 4.1. Collection of Wheat Samples and Isolation of Fusarium Strains

A total of 141 durum wheat samples were collected from three different Italian geographical macro-areas, Northern, Central and Southern Italy, during the period 2013–2015.

In particular, 40, 64 and 37 samples were collected in 2013, 2014 and 2015, respectively, from commercial fields located in Northern Italy (Piemonte, Veneto and Emilia Romagna regions), Central Italy (Marche) and Southern Italy (Molise, Puglia, Basilicata, Campania and Sicilia), as reported in Figure S1. In 2013, wheat samples were collected only from Central and Southern Italy regions. From each field, wheat plants were collected by using the X-shaped sampling method. Approximately 1 kg of kernels for each sample was then collected and divided into two homogeneous kernel subsamples of 0.5 kg each to be used for seed mycological analysis and chemical analyses. In order to detect the fungal population present in each wheat sample, one-hundred kernels were properly surface disinfected and plated on PDA medium supplemented with PCNB (pentachloronitrobenzene) and streptomycin and neomycin sulphate antibiotics, as reported by Masiello et al. [46]. After 5 days of incubation at 25 °C, the number of kernels developing colonies of *Alternaria, Aspergillus*, *Penicillium* and *Fusarium* species were counted. Contamination values were calculated as the number of contaminated kernels on total analyzed kernels, expressed as a percentage.

### 4.2. Fusarium Species Identification

A representative colony of each morphotype was single spored on PDA substrate and transferred on CLA (Carnation Leaf Agar) and SNA (synthetic nutrient-poor agar) media for the morphological identification at the species level according to Leslie and Summerell [13].

To confirm species identification, selected strains belonging to different *Fusarium* species according to morphological identification, were molecularly identified by sequencing the 1-α translation elongation factor (*TEF*) gene. The same selected strains were stored in the ITEM fungal collection of the Institute of Sciences of Food Production, ISPA (http://www.ispa.cnr.it/Collection (accessed on 7 September 2022)).

The strains selected for molecular analyses were cultured on cellophane disks overlaid on PDA plates and DNA was extracted from10 mg of lyophilized fungal biomass by using Wizard^®^ Magnetic DNA Purification System kit for Food (Promega), as reported by Noorabadi et al. [47].

The elongation factor gene was selected as the most common informative marker used for *Fusarium* identification at the species level. For each strain, a fragment of the TEF gene was amplified with primer pair EF1/EF2 from O’Donnell et al. [48] in 15 μL volume containing 1× HotMaster Taq DNA Polymerase Buffer (5-PRIME), 200 µM dNTPs, 300 nM each primer, 0.375 U of T HotMaster Taq DNA Polymerase (5-PRIME) and 25 ng of genomic DNA. The PCR conditions were 94 °C for 2 min, followed by 35 cycles of 94 °C for 50 s, 59 °C for 50 s and 72 °C for 1 min, and a final step at 72 °C for 5 min. The size of PCR amplicons were determined on 1.5% agarose gel.

The PCR products, purified by an enzymatic method (FastAP Thermosensitive Alkaline Phosphatase and Exonuclease I; Thermo Scientific, Waltham, MA, USA), were sequenced for both strands by using the Big Dye Terminator v 3.1 Cycle Sequencing Kit (Thermo Scientific, Waltham, MA, USA), according to the manufacturer’s instructions. The labeled fragments were then purified by gel filtration through mini-columns containing Sephadex G-50 (Sigma-Aldrich, Milan, Italy), denatured for 5 min at 95 °C and run by capillary electrophoresis on the 3730 × l DNA Analyzer (Applied Biosystems, Foster City, CA, USA). DNA sequences were analyzed with the Sequencing Analysis 5.2 software (Applied Biosystems, Waltham, MA, USA) and consensus sequences were obtained aligning both strands with Bionumerics software (Applied Maths, Kortrijk, Belgium). All sequences were compared among them and to available sequences in GenBank by using the Basic Local Alignment Tool (BLAST).

### 4.3. Chemical Analyses

#### 4.3.1. Chemicals and Reagents

All solvents (HPLC grade) were purchased from VWR International Srl (Milan, Italy). DON, NIV, T-2 and HT-2 toxins and ZEA of an analytical standard grade (purity > 99%) were supplied by Sigma-Aldrich (Milan, Italy). Immunoaffinity columns were provided from VICAM L.P. (Milford, MA, USA); glass microfiber filters and paper filters from Whatman (Maidstone, UK).

#### 4.3.2. Sample Preparation

All kernels of each subsample (0.5 kg) were milled and homogenized using an ultra-centrifugal mill (ZM 200, Retsch) equipped with a 500 µm sieve. After milling and homogenization, the samples were stored at 4 °C until use. Chemical analyses were carried out on homogeneous aliquots of 25 g for each wheat sample.

#### 4.3.3. Analysis of T-2 and HT-2 Toxins

Wheat samples were extracted with methanol/water (90:10, *v*/*v*) and diluted extracts were cleaned up through immunoaffinity columns. T-2 and HT-2 toxins were separated and quantified by Ultra Performance Liquid Chromatography (UPLC) with a photodiode array (PDA) detector (λ = 202 nm). The retention time of HT-2 and T-2 toxins was 1.97 and 4.9 min, respectively. The mycotoxins were quantified by comparing peak areas with calibration curves obtained with standard solutions. The linearity of the analytical response was determined by analyzing the calibration standards and using seven concentrations over the range 62.5–4000.0 ng/mL of T-2 and HT-2 toxins. The limit of detection (LOD) of the method was 8 µg/kg for both toxins (signal to noise 3:1) [49].

#### 4.3.4. Analysis of DON and NIV

Wheat samples were extracted with water and the filtered extracts were cleaned up through an immunoaffinity column containing a monoclonal antibody specific for DON and NIV. Toxins were separated and quantified by UPLC with a PDA detector (λ = 220 nm). The retention time of NIV and DON was 1.4 and 2.4 min, respectively. The linearity of the analytical response was determined by analyzing the calibration standards and using eight concentrations over the range 10.0–5000.0 ng/mL of NIV and DON. The LOD of the method was 20 µg/kg for NIV and DON (signal to noise 3:1) [50].

#### 4.3.5. Analysis of ZEA

Wheat samples were extracted with acetonitrile-water (90:10, *v*/*v*) and the extract was diluted with water (1:10, *v*/*v*) and applied to an immunoaffinity column. The column was washed with water and ZEA was eluted with methanol and quantified by reversed-phase HPLC with fluorometric detection (λ ex = 274 nm, λ em = 440 nm) using acetonitrile-water-methanol (46:46:8, *v*/*v*) as the mobile phase. The retention time was 7.2 min. The linearity of the analytical response was determined by analyzing the calibration standards and using six concentrations over the range 10.0–300.0 ng/mL of ZEA. The LOD of the method was 3 µg/kg for ZEA (signal to noise 3:1) [51].

## 5. Conclusions

The present paper analyzes and discusses data of *Fusarium* contamination and related mycotoxins in Italian durum wheat collected in the period 2013–2015 from fields located from Northern to Southern Italy. This study provides a clear picture of durum wheat contamination associated with FHB in a missing period in the literature. High variability was observed depending on geographical areas and sampling years, although the prevalence of DON, with *F. graminearum* as the main occurring species, has been observed in Northern Italy, while high contamination by T-2 and HT-2 toxins and the presence of *F. sporotrichioides* and *F. langsethiae* have been detected in Southern regions. Moreover, in several samples, mycotoxin values were over the EU established limits, representing a great concern for Italian durum wheat. The importance of constantly monitoring *Fusarium* contamination and related mycotoxin occurrence has therefore been underlined.

## Figures and Tables

**Table 1:** Statistical data of mycotoxin-contaminated Italian wheat samples in different geographical areas in three years (2013–2015). For each mycotoxin, deoxinivalenol (DON), nivalenol (NIV), and zearalenone (ZEA), the sum of T-2 and HT-2 toxins and the number and percentage of positive samples are shown. Mean, median and range values, expressed in µg/kg, were calculated on positive samples.

		Northern Italy				Central Italy				Southern Italy			
		DON	NIV	ZEA	T-2 + HT-2	DON	NIV	ZEA	T-2 + HT-2	DON	NIV	ZEA	T-2 + HT-2
2013	N. of positive samples/total	-	-	-	-	7/20	-	-	12/20	0	1/20	19/20	20/20
	% positive samples	-	-	-	-	35	-	-	60	0	5	95	100
	Mean	-	-	-	-	376	-	-	40	0	200	53	154
	Median	-	-	-	-	80	-	-	21	0	200	24	145
	Maximum	-	-	-	-	2230	-	-	123	0	200	297	335
	Range	-	-	-	-	20–2230	-	-	8–123	0	200	4–297	10–335
	N. of samples exceeding legal limits ^1^	-	-	-	-	1	-	-	2	0	-	3	17
2014	N. of positive samples/total	7/12	0/12	5/12	2/12	5/28	2/28	9/28	17/28	0/24	0/24	20/24	20/24
	% positive samples	58	0	42	17	18	7	32	61	0	0	83	83
	Mean	462	0	9	11	2227	56	24	28	0	0	49	310
	Median	519	0	6	11	469	56	31	15	0	0	20	336
	Maximum	966	0	23	13	9129	71	54	68	0	0	325	486
	Range	58–966	0	3–23	8–13	112–9129	40–71	3–54	10–68	0	0	4–325	35–486
	N. of samples exceeding legal limits	0	-	0	0	1	-	0	0	0	-	3	18
2015	N. of positive samples/total	1/6	1/6	3/6	0/6	0/20	0/20	0/20	0/20	0/11	0/11	4/11	6/11
	% positive samples	17	17	50	0	0	0	0	0	0	0	36	54
	Mean	2500	76	6	0	0	0	0	0	0	0	5	28
	Median	2500	76	6	0	0	0	0	0	0	0	5	23
	Maximum	2500	76	9	0	0	0	0	0	0	0	5	60
	Range	2500	76	4–9	0	0	0	0	0	0	0	4–5	12–60
	N. of samples exceeding legal limits	1	-	0	0	0	-	0	0	0	-	0	0

^1^ EU legal limits have not been established for nivalenol.

**Table 2:** List of *Fusarium* species considered as producers of the mycotoxins deoxynivalenol (DON), nivalenol (NIV), zearalenone (ZEA), T-2 and HT-2 toxins (T-2/HT-2), diacetoxyscirpenol (DAS), monoacetoxyscirpenol (MAS), neosolaniol (NEO), beauvericin (BEA), enniatins (ENNs), moniliformin (MON), fumonisins (FUM).

Mycotoxins	Producing *Fusarium* Species
DON	*F. graminearum*, *F. culmorum*, *F. crookwellense*
NIV	*F. graminearum*, *F. culmorum*, *F. crookwellense*, *F. poae*, *FIESC*
ZEA	*F. graminearum*, *F. culmorum*, *F. crookwellense*, *FIESC*
T-2/HT-2	*F. sporotrichioides*, *F. langsethiae*
DAS, MAS, NEO	*F. poae*, *F. avenaceum*, *F. tricinctum*, *F. acuminatum*, *FIESC*
BEA	*F. poae*, *F. avenaceum*, *F. acuminatum*, *F. proliferatum*, *F. oxysporum*
ENNs	*F. poae*, *F. chlamydosporum*, *F. avenaceum*, *F. acuminatum*, *F. oxysporum*
MON	*F. chlamydosporum*, *FIESC*, *F. proliferatum*, *F. subglutinans*, *F. oxysporum*, *F. solani*
FUM	*F. verticillioides*, *F. proliferatum*

## Data Availability

Not applicable.

## Supplementary Materials

The following supporting information can be downloaded at: https://www.mdpi.com/article/10.3390/toxins14090627/s1, Figure S1: Distribution of wheat sampling in Italian regions in cropping seasons 2013–2015.
