# Supplementary figures and images for: Deoxynivalenol and T-2 Toxin as Major Concerns in Durum Wheat from Italy
Source: Toxins (Basel). 2022 Sep 8;14(9):627. doi: 10.3390/toxins14090627 (PMC9503377; doi:10.3390/toxins14090627)

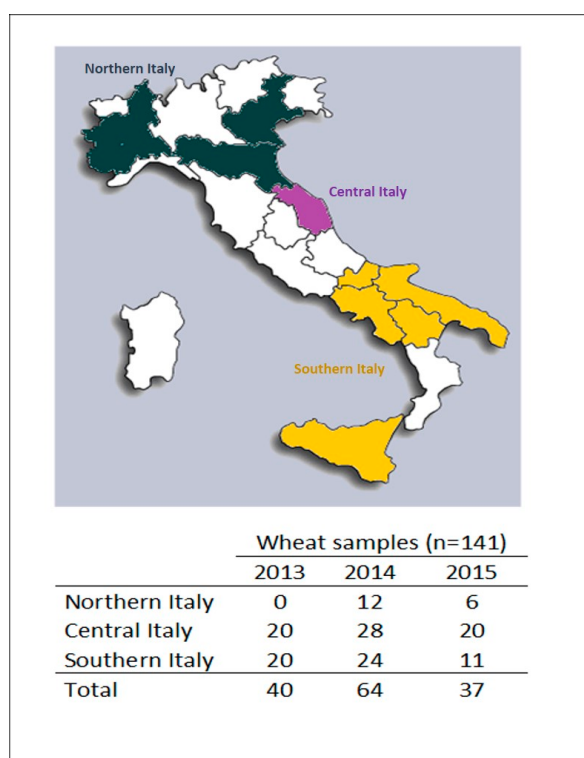

**Figure S1.** Distribution of wheat sampling along Italian regions in cropping seasons 2013-2015.

Supplement: Supplementary file 1 [file toxins-14-00627-s001.zip › toxins-1849855-supplementary.pdf]
